# Thiazides in the management of hypertension in older adults – a systematic review

**DOI:** 10.1186/s12877-017-0576-3

**Published:** 2017-10-16

**Authors:** Christina Sommerauer, Neha Kaushik, Adrine Woodham, Anna Renom-Guiteras, Yolanda V Martinez, David Reeves, Ilkka Kunnamo, Thekraiat Al Qur‵an, Steffen Hübner, Andreas Sönnichsen

**Affiliations:** 1 Institute of General Practice and Family Medicine, University of Witten/Herdecke, Alfred-Herrhausen-Straße 50, 58448 Witten, Germany; 2 University of Manchester, Centre for Primary Care, Institute of Population Health, Manchester, UK; 3 Department of Geriatrics, University Hospital Parc de Salut Mar, Barcelona, Spain; 4 NIHR School for Primary Care Research, Manchester Academic Health Science Centre, University of Manchester, Manchester, England England; 5 Duodecim Medical Publications Ltd, Helsinki, Finland; 6 Department of Public Health, Community Medicine, Jordan University of Science and Technology, Irbid, Jordan

**Keywords:** Older adults, Thiazides, Hypertension, Systematic review, Polypharmacy

## Abstract

**Background:**

Thiazides are commonly prescribed to older people for the management of hypertension. The objective of this study was to identify the evidence on the risks and benefits of their use among adults aged ≥65 years and to develop recommendations to reduce potentially inappropriate use.

**Methods:**

Systematic review (SR) of the literature covering six databases. We applied a staged search approach, where each search was undertaken only if the previous one did not yield high quality results. Searches 1 and 2 identified relevant SRs and meta-analyses published up to December 2015 from all databases. Search 3 identified additional individual interventional studies (IS) and observational studies (OS) not identified by the preceding searches. We included all studies evaluating the effect of thiazides on patient-relevant outcomes in the management of hypertension with a sufficient number of participants aged ≥65 years or a subgroup analysis based on age. Two independent reviewers extracted data and carried out quality appraisal. Recommendations were developed using the GRADE methodology.

**Results:**

Searches 1 to 3 were performed. We included 34 articles reporting on 12 IS and 4 OS. Mean ages ranged from 59 to 83.8 years. Four studies had performed a subgroup analysis by age. Information on comorbidity, polypharmacy and frailty of the participants was scarce or not available. The IS compared thiazides to placebo or other antihypertensive drugs and evaluated cardiovascular endpoints or all-cause-mortality as primary outcomes. The OS investigated the association between thiazide use and the risk of gout, fractures and adverse effects. Our results suggest that thiazides are efficacious in preventing cardiovascular events for this population group. Low-dose regimens of thiazides may be safer than high-dose (low quality of evidence), and a history of gout may increase the risk of adverse events (low quality of evidence). Three recommendations were developed.

**Conclusions:**

The use of low dose treatment with thiazides for the management of hypertension in adults aged 65 and older seems justified, unless a history of gout is present. The quality of the evidence is low and studies rarely describe characteristics of the participants such as polypharmacy and frailty. Further good quality studies are needed.

**Electronic supplementary material:**

The online version of this article (doi:10.1186/s12877-017-0576-3) contains supplementary material, which is available to authorized users.

## Background

Hypertension is highly prevalent worldwide and associated with increased morbidity for cardiovascular diseases. The incidence of hypertension increases with age. In the Framingham cohort in the US, nearly 75% of participants aged 80 years or older were hypertensive, and more than 60% had stage 2 hypertension [1]. Older people (≥ 60 years) benefit from antihypertensive treatment because it reduces mortality and the incidence of stroke and myocardial infarction [2]. These benefits can also be seen in very old patients, but less clearly in frail older people with multimorbidity and polypharmacy [3].

The group of thiazides is one of the key medications used in the management of hypertension. In the European Society of Hypertension (ESH)/European Society of Cardiology (ESC) guidelines, all types of antihypertensive drugs are recommended for older patients, although diuretics and calcium channel blockers are preferred for isolated systolic hypertension [4]. Similarly, in the updated Eighth Joint National Committee guidelines (JNC-8) thiazides are equally recommended together with calcium channel blockers, ACE inhibitor or angiotensin receptor blockers [5]. Most guidelines like JNC-8 or the ESH/ESC guidelines do not differentiate between thiazide diuretics and thiazide-like diuretics like chlorthalidone. Furthermore, hydrochlorothiazide (HCT) has been considered equivalent to chlorthalidone due to the same mechanism of action [4, 5]. On the other hand, the NICE guideline for hypertension explicitly recommends thiazide-like diuretics in preference to a conventional thiazide [6]. It is not clear which thiazide is most preferable, especially for older adults.

Beside the known benefits mentioned above, treatment with thiazides has been linked to adverse events such as electrolyte imbalances and glucose intolerance [7]. As a result of renal insufficiency, which occurs more often in older age, older people are at higher risk of these adverse drug events. A study of drug related emergency department visits by older people showed an association between thiazide use and drug related problems that can lead to hospitalization [8]. The published STOPP and START criteria judged thiazides as potentially inappropriate for older adults with gout [9].

Concerns about the use of thiazides in older people may be justified because recommendations in clinical guidelines are often based on evidence from younger populations, as older people are frequently excluded from high quality clinical trials [10].

To the best of our knowledge, there is no existing systematic review (SR) evaluating the efficacy and safety of thiazides explicitly for older people. The objectives of this review were:

- To systematically identify the best available evidence on the risks and benefits of treatment with thiazides in hypertensive older patients, especially in those with comorbidity and polypharmacy
- To critically assess the quality of this evidence
- To develop recommendations for older adults with hypertension when to stop, switch to another antihypertensive medication or decrease the dose of thiazides.

These recommendations will be incorporated into an electronic decision support tool which has been developed for the PRIMA-eDS project (Polypharmacy in chronic diseases: Reduction of Inappropriate Medication and Adverse drug events in older populations by electronic Decision Support) and will be used by General Practitioners to reduce polypharmacy [11].

## Methods

We performed a systematic review (SR) of the literature as part of a set of SRs carried out for the EU project PRIMA-eDS. A detailed description of the methods used can be found in this special issue. The methods used are based on the methodological manuals of the Cochrane collaboration [12] and the PRISMA statement for reporting SRs [13, 14]. We carried out the SR according to a piloted protocol template and standard operating procedures, as set out in a specific SR study protocol (available upon request). A team of 6 trained reviewers participated (CS, AW, NK, SH, YVM, ARG).

### Literature search

We followed a staged search approach consisting of four sequential literature searches. Each search was performed if the preceding search did not yield high quality results or if the research team decided that the evidence identified was insufficient to enable an evidence based recommendation to be made. The searches included the following databases and types of studies:

- Search 1: Systematic reviews and meta-analyses in the Cochrane database of Systematic Reviews (from 2005 onwards) and the Database of Abstracts or Reviews of Effects (DARE, from 1991 onwards)
- Search 2: Systematic reviews and meta-analyses in MEDLINE (from 1946 onwards), EMBASE (from 1974 onwards), Health Technology Assessment (HTA, from 2001 onwards) and International Pharmaceutical Abstracts (IPA, from 1970 onwards)
- Search 3A: Interventional and observational studies eligible by themselves, taken from systematic reviews that as a whole failed the inclusion criteria for searches 1 and 2
- Search 3B: Additional controlled interventional and observational studies identified from MEDLINE (from 1946 onwards), EMBASE (from 1974 onwards), HTA (from 2001 onwards) and IPA (from 1970 onwards)

A search strategy was developed according to the PICOS framework. Additional file 1 presents the search strategy used for this SR. The searches were performed in December 2015. No language restrictions were applied to the search but only studies in English, German, Finish, Italian and Spanish were considered for inclusion.

Additionally, we checked the reference lists of included articles to identify further articles for inclusion. Where we identified further articles the reference lists of these were also checked. We also considered studies identified from manual searches, e.g. articles identified from other reviewers participating in other SRs of the set of SRs or articles identified by experts.

### Study selection

Two reviewers independently performed study selection according to predefined inclusion and exclusion criteria (Table 1). Abstracts and titles were screened for inclusion criteria and any differences in opinion between the two reviewers were resolved by discussion or if necessary by arbitration involving a third reviewer. Subsequently, full texts were reviewed for inclusion utilising the same procedure.

**Table 1:** Inclusion and exclusion criteria

Inclusion criteria	Exclusion criteria
• Systematic reviews and meta-analyses: mean age ≥ 65 years • Original studies: at least 80% of participants ≥65 years old OR • Subgroup analysis reporting on participants ≥65 years • Intervention: thiazide or thiazide-like diuretics • Condition: hypertension • Clinical relevant outcomes	• Studies focusing only on acute/short term conditions • Studies evaluating only surrogate endpoints (like blood pressure) • The following publication types: ⋅ Editorials ⋅ Opinion papers ⋅ Case reports, case series ⋅ Narrative reviews ⋅ Letters ⋅ Qualitative studies

Systematic reviews were defined as reviews with a systematic literature search and a systematic study selection in accordance with widely accepted methodology like e.g. the Cochrane Handbook for Systematic Reviews [12].

### Data extraction and quality appraisal

Data extraction was carried out by one reviewer for all included articles using a piloted data extraction form specific for each study type and checked by the second reviewer for accuracy and completeness.

Quality appraisal was performed by the one reviewer for each included article and checked by another reviewer. Quality appraisal was evaluated by means of validated tools specific for each study type: for systematic reviews we used the tool for the assessment of multiple systematic reviews (AMSTAR) [15, 16]; for clinical trials we used the Cochrane Handbook for Systematic Reviews of Interventions [12]; for observational studies we used a selection of questions extracted from the Critical Appraisal Skills Programme (CASP) [17, 18].

### Identification of references of interest for the development of recommendations

Reviewers identified additional references that did not meet the inclusion criteria for the SR but were considered of interest for the development of recommendations. These references were identified during the searches, the handsearch or by checking the reference lists of the included articles. References of interest mostly related to younger populations or were clinical guidelines or provided the review team with additional evidence about thiazides.

### Development of recommendations

Reviewers developed recommendations during team meetings based on the results of the included studies and the additional references of interest using the Grading of Recommendations Assessment, Development and Evaluation (GRADE) methodology [19–21]. We aimed at developing recommendations that focused on stopping a drug or reducing its dose, as these recommendations could be used to reduce polypharmacy. The recommendations followed a standard wording according to their strength (weak or low) and the quality of their evidence (low, moderate or high). For reason of simplification we used only three categories for the quality of evidence, following the American College of Physicians’ Guideline Grading System [22]. The suggestions for recommendations were discussed and approved by an editorial board for the development of evidence based medicine (EBM) guidelines and recommendations of Duodecim Medical Publication Ltd. from Finland. The Editorial Team of the EBMeDS decision support service includes physicians and nurses and finalizes the decision support rules. Four out of 10 members are also members of the EBM Guidelines Editorial Team or Editorial Board. Four other members of the EBM Guidelines Editorial Team serve as advisors and referees for EBMeDS contents. The members of the teams do not have conflicts of interests [23]. The recommendations will be implemented in an electronic decision support tool which is currently being tested by General practitioners in a large clinical trial [11].

### Data synthesis

Results of the included studies were summarised in a narrative synthesis reporting on the participants, types of outcomes and quality of the included studies. A meta-analysis was not performed due to high heterogeneity of the included studies which investigated different types of thiazides and varied in population and comparison groups.

## Results

**Fig. 1:**
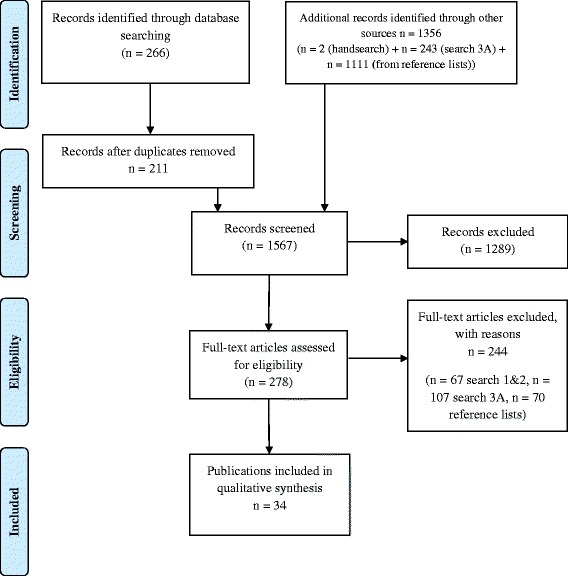
Preferred Reporting Items for Systematic Reviews and Meta-Analyses (PRISMA) flow diagram

Figure 1 displays the process of identification of studies for inclusion in the systematic review. Searches 1, 2 and 3A were performed. The research team decided not to perform Search 3B because the evidence already identified was sufficient for the preparation of the recommendations and it was not expected that a further search would provide evidence to substantially alter the results. We identified 266 references from database searching (search 1 and 2) and additionally 243 references from search 3A and 1111 from the reference lists of included articles. Two articles were identified by hand search. After removing duplicates, we screened 1567 records and assessed 278 full texts. A list of excluded studies along with the reasons for exclusion is available upon request. The main reasons for exclusion were insufficient participants aged 65 or over, or that systematic reviews were not focused on thiazides.

No relevant systematic reviews fulfilling our inclusion criteria were identified, but 34 articles relating to 16 unique individual studies published between 1974 and 2012 were included [24–57]. Thirty-two references were identified from search 3A and two references from the handsearch. The characteristics of the included studies and the corresponding articles are shown in Additional file 2: Table S1. Eleven studies were randomized controlled trials, one study was a non-randomized prospective clinical trial and four studies were observational studies (two retrospective cohort studies and two case control studies). The mean age of participants in the studies ranged from 59 to 83.8 years, but three studies were included based on availability of a subgroup analysis for patients 65 years or older [24, 36, 40].

Additional file 3: Table S2 presents the characteristics of the participants in each study which was mostly limited to comorbidity, sex and age. The stage of hypertension according to JNC-7 or ESC Guidelines often was not reported, but summary blood pressure information was usually provided. None of the trials reported the number of patients with polypharmacy and information about frailty level and cognitive status was scarce.

Different types of thiazides were studied. Four trials used chlorthalidone as the study drug, three hydrochlorothiazide, and two indapamide. Bendroflumethiazide, methylclothiazide and trichlormethiazide were used in one trial each. Four randomized trials used a fixed combination therapy including a thiazide as a starting drug. Three of the four observational studies did not focus on a specific type of thiazide and included any thiazides. One observational study compared hydrochlorothiazide and chlorthalidone. Seven trials compared thiazides to placebo, one to no treatment. In three trials thiazides were compared to a calcium channel blocker, one of these was a subgroup analysis. In addition one trial compared thiazides to β-blocker in a subgroup analysis.

Clinically relevant outcomes reported in the included studies were cardiovascular morbidity and

mortality including stroke as single outcome measure, all-cause mortality, fractures and adverse

events.

### Effects of the use of thiazides in the management of hypertension in older people

For each study and outcome Additional file 4: Table S3 summarises the results for the thiazide and comparison groups, provides estimated risk ratios with 95% confidence intervals, and reports any statistical comparisons of the study itself. To help interpretation, Additional file 4: Table S3 organises the results by type of outcome. The results for Dhalla et al. [29] are not represented in the table as this observational study compares two different thiazides. We did not identify any study reportin on other outcomes that were covered by our search strategy such as falls or hospitalisation.

#### Cardiovascular outcomes

Most trials reported on cardiovascular outcomes. Nine trials reported on cardiovascular outcomes as the primary outcome.

#### Stroke

Four trials evaluated fatal and non-fatal stroke as the primary outcome [36, 37, 44, 49] with placebo as the comparison and two trials stroke as a secondary outcome against other active treatments [24, 43]. Study drugs included different types of thiazides. Two large-scale trials showed a significant advantage for thiazides in the reduction of stroke in comparison to placebo [44, 49]. In addition, a significant reduction of stroke was seen in the SHEP pilot trial which compared chlorthalidone to placebo [47]. The Hypertension in the Very Elderly Trial (HYVET) reported a 30% reduction for indapamide compared to placebo in the rate of stroke which did not quite reach statistical significance [37]. The HSCS trial found a non-significant 25% reduction of stroke events in the 65 and older subgroup for deserpidine plus methylchlothiazide compared to placebo but the sample size was small [36]. Across the full study sample of this trial (stroke survivors with a mean age of 59 years) there was no difference in the occurrence of stroke (including transient ischemic attacks). In comparison to other active treatments (lisinopril, amlodipine, lacidipine, atenolol) in three RCTs [24, 43, 44], the only significant advantage regarding the risk of stroke was for chlorthalidone compared to lisinopril, reported for the subgroup analysis for patients aged ≥65 years from the ALLHAT trial [24].

#### CVD/CHD

Two trials reported on CVD and CHD [24, 49]. Chlorthalidone was associated with lower risk for CVD and CHD compared to placebo [49] and compared to lisinopril and doxazosin [24].

#### Heart failure

Two trials reported on heart failure [24, 49]. In the SHEP trial [49], non-fatal heart failure occurred less often in the chlorthalidone than in the placebo group. In the ALLHAT trial [49], heart failure was less frequent with chlorthalidone compared to other active treatments (amlodipine, lisinopril, doxazosin).

#### Combined cardiovascular/cerebrovascular endpoints

Ten studies reported on composite endpoints of cardiovascular morbidity/mortality events, four trials [24, 40, 41, 43] and one observational study [29] as primary outcome and five RCTs as secondary outcome [31, 36, 44, 48, 49]. The specific events making up the composite endpoint varied markedly across studies.

Four trials showed a significant advantage for thiazides compared to placebo: The SHEP trial showed benefits for chlorthalidone compared to placebo regarding nonfatal MI or coronary heart disease (CHD)(see Additional file 4: Table S3) [49]. The SHEP pilot study showed significantly less hypertensive events associated with chlorthalidone than with placebo but no significant difference for all atherosclerotic events [46]. The EWPHE trial showed significantly less non-fatal cardiovascular study terminating events for HCT/triamterene compared to placebo [31]. In the MRC-O trial, coronary events occurred less often in the diuretic group compared to the placebo group [44]. Three studies compared thiazides to other active treatments and used a composite of cardiovascular outcomes as primary outcome.

In the ACCOMPLISH trial, the combination of an ACE-inhibitor with a calcium channel blocker was more effective for a composite of cardiovascular endpoints than the combination of an ACE-inhibitor with hydrochlorothiazide, in the subgroups for patients ≥65 years and ≥70 years [40]. In the ALLHAT trial, in participants aged ≥65 years chlorthalidone showed the same advantage regarding a composite of fatal CHD and non-fatal MI compared to both amlodipine and lisinopril. Results on the primary outcome for the older subgroup were not presented for the groups using doxazosin and ACE-inhibitors, but for all participants chlorthalidone showed less cardiovascular events than doxazosin, which led to a premature closing of the doxazosin arm [24, 26].

The SHELL study compared chlorthalidone to the calcium channel blocker lacidipine and found no significant difference regarding cardiovascular morbidity and mortality [43].

In addition, the MRC-O trial showed a significantly lower rate of coronary events (as a secondary endpoint) amongst patients on hydrochlorothiazide compared to those taking β-blockers [44].

Two different types of thiazide, chlorthalidone and HCT, were compared against each other by Dhalla et al. [29] in a large observational study of 30,000 patients. No significant difference was found on a composite endpoint of death or hospitalization for heart failure, stroke or myocardial infarction.

#### Mortality

Ten studies reported on all-cause mortality and one trial, the EWPHE trial, analyzed all-cause mortality as the primary outcome [24, 29, 31, 37, 39, 41, 43, 44, 48, 49]. Comparison groups included no treatment, placebo and other antihypertensive drugs. The HYVET trial showed a significant 21% reduction of all-cause mortality for the indapamide group vs. placebo. In all other trials there was no significant benefit or risk regarding all-cause mortality for the thiazide-treatment groups.

Cardiovascular mortality did not appear as a primary outcome in any study, but was analyzed as a secondary outcome in four trials that compared thiazides to placebo [31, 37, 39, 44]. The EWPHE and the MRC-O trials showed a significant reduction of cardiovascular mortality in the diuretic treatment group [31, 44]. In the EWPHE trial, cardiovascular mortality was significantly reduced in the HCT group compared to placebo. In a secondary analysis, the effect of treatment was negatively associated with age, and in the subgroup aged >80 years no effect was observed [30]. The HYVET trial and the HYVET pilot demonstrated no significant benefit regarding cardiovascular mortality for indapamide and bendroflumethiazide compared to placebo/no treatment [37, 39].

The MRC-O trial additionally compared HCT to atenolol and found significantly fewer cardiovascular deaths occurred in the hydrochlorothiazide group compared to the β-blocker group. The two treatment groups combined showed no reduction in cardiovascular mortality compared to placebo [44].

#### Adverse events

Two trials reported on serious adverse events (SAE) using thiazides compared to placebo [28, 37]. In the HYVET trial, serious adverse events occurred significantly less often in the indapamide treatment group than in the placebo group, and only five SAEs were judged to be related to study medication (3 in the placebo group, 2 in the indapamide group) [37]. A RCT that compared the combination of perindopril/indapamide to placebo reported that two SAEs possibly related to study medication occurred in each group [28].

The SHEP trial reported that the prevalence of intolerable symptoms was higher in the chlorthalidone group compared to placebo, and the EWPHE trial found three symptoms significantly more common in patients on hydrochlorothiazide compared to placebo (dry mouth, nasal stuffiness and diarrhea) but none to the opposite. The EWPHE trial also reported that significantly more treated patients stopped the study medication because of side effects or concomitant disease compared to those taking placebo [32, 57]. Likewise, in the MRC-O trial withdrawals due to major side effects were considerably higher in the diuretic group than in the placebo group, partly due to increased gout, but also to significantly greater incidence of impaired glucose tolerance, skin disorders, muscle cramp, nausea and dizziness.

Less evidence was available for comparing rates of adverse events on thiazides to other antihypertensive drugs. In the SHELL study, chlorthalidone was compared to lacidipine: fatigue occurred significantly more often in the chlorthalidone group, but edema (mainly pretibial), headaches and skin rashes significantly more often in the calcium channel blocker group [43]. Other side effects were similar in both groups. In the MRC-O trial, withdrawals due to major side effects were less frequent in the thiazide group than in the β-blocker group, although gout and muscle cramp were more common. In the SHEP trial cases of new-onset diabetes did not differ between chlorthalidone and placebo groups at years one and three [54]. Finally, the ACCOMPLISH trial found no increased risk of SAE associated with benazepril/HCT treatment compared to benazepril/amlodipine across the main study population with a mean age of 68 years [40].

#### Gout

A retrospective cohort study investigated the risk of initiation of anti-gout therapy in hypertensive patients and found a higher risk for patients exposed to thiazides increasing with higher doses. No increased risk could be seen at doses <25 mg/dl [35], but the wide confidence interval did not exclude a possible association. This study agrees with the findings in the EWPHE trial in which reports of gout were significantly more frequent in the HCT treated group [31] and the MRC-O trial with high numbers of study medication withdrawal due to gout [44].

#### Fractures

Two observational studies investigated a potential protective effect of antihypertensive treatment with thiazides on the incidence of hip fracture [42, 56]. A case control study with female participants found no difference in the risk of hip fracture between thiazide users and non-users for either current or former thiazide users [56]. In contrast, a prospective case control study with 9518 patients reported a considerably lower incidence of hip fracture among thiazide users compared to non-users [42].

#### Other outcomes

No association between thiazide use and the occurrence of new-onset diabetes [54], dementia [38, 45] or depression [45] was found in the included studies.

### Additional references for the development of recommendations

Three additional references were identified as being of interest for the development of recommendations [9, 58, 59]. The STOPP list [9] and the American College of Rheumatology Guidelines for Management of Gout [59] supported the evidence we found on gout. The systematic review by Wright et al. [58] reported on morbidity and mortality for different antihypertensive drugs including thiazides. Thiazides were associated with a reduction in stroke, coronary disease, cardiovascular events and mortality. High doses of thiazides only reduced stroke and total cardiovascular events, but not mortality or coronary artery disease.

### Risk of bias

Tables 2 and 3 present the results of quality appraisal of the clinical trials and observational studies, respectively. The quality of the clinical trials was judged to be mostly low to moderate, with only one study judged to have high quality. At least one quality item was not clearly reported in all the observational studies.

**Table 2:** Results of quality appraisal of the interventional studies according to the Cochrane Handbook of Interventions

Source	Type of study	Selection bias		Performance bias	Detection bias	Attrition bias	Reporting bias	
		1. Random sequence generation	2. Allocation concealment	3. Blinding of participants and personnel	4. Blinding of outcome assessment	5. Incomplete outcome data	6. Selective reporting	7. Other bias
ACCOMPLISH Jamerson et al. 2008 [40]	Multicentre trial in US and North Europe	UR	LR	LR	LR	LR	UR	UR
ALLHAT 2002 [24]	double blind RCT	LR	LR	LR	LR	LR	LR	UR
Chalmers et al. 2000 [28]	double blind RCT	UR	UR	LR	LR	LR	LR	UR
EWPHE Amery et al. 1985 [31], Fletcher 1991 [32], Staessen et al. 1989 [33] Staessen et al. 1991 [34]	double blind RCT	LR	UR	LR	LR	HR	LR	UR
HSCS 1974 [36]	double blind RCT	UR	UR	LR	UR	UR	LR	HR
HYVET pilot Bulpitt et al. 2003 [39]	RCT	LR	LR	HR	HR	LR	LR	LR
HYVET Beckett et al. 2008 [37]	double-blind RCT	LR	LR	LR	LR	UR	LR	LR
Kuramoto et al. 1981 [41]	Prospective clinical trial	UR	UR	UR	UR	HR	LR	HR
MRC-O trial 1992 [44]	single blind RCT	UR	UR	LR	LR	HR	UR	HR
SHELL Malacco et al. 2003 [44]	RCT open design	LR	UR	HR	HR	HR	LR	HR
SHEP pilot Hulley et al. 1985 [46] Perry et al. 1986 [47] Perry et al. 1989 [48]	double-blind RCT	LR	UR	LR	LR	HR	UR	UR
SHEP SHEP Group 1991 [49], Hawkins 1993 [50], Perry et al. 2000 [51], Kostis et al. 1997 [52], Curb et al. 1996 [53], Savage et al. 1998 [58], Somes et al. 1999 [55]	double-blind RCT	LR	LR	LR	LR	LR	LR	UR

*LR* low risk of bias, *HR* high risk of bias, *RCT* Randomized controlled trial, *UR* unclear risk of bias

**Table 3:** Results of the quality appraisal of the observational studies according to CASP

Source	Focused issue	Appropriate method	Recruitment	Selection of controls	Exposure measured	Outcome measured	Identified confounding factors?	Confounding factors in design/analysis	Follow up complete	Follow up long	Believe results	Results be applied	Results fit evidence
Dhalla et al. 2013 [29]	Y	Y	Y	Y	U	Y	Y	Y	Y	Y	Y	U	N
Gurwitz et al. 1997 [35]	Y	Y	Y	Y	N	N	Y	Y	U	Y	Y	N	Y
LaCroix et al. 1990 [42]	Y	U	Y	Y	N	U	Y	Y	U	U	Y	U	N
Weiland et al. 1997 [56]	Y	U	Y	Y	U	U	N	N	Y	Y	U	Y	U

*Y* yes, *N* no, *U* unclear, *NA* not applicable

### Development of recommendations

Based on the evidence identified and the additional references of interest we developed three recommendations which are presented in Additional file 5: Table S4. All recommendations were considered to have a weak strength and low quality of evidence. Additional file 5: Table S4 displays the reasons for the strength and quality of evidence, and the main articles which constitute the evidence base for each recommendation, although all included studies were taken into account for the risk/benefit balance during the team meetings.

We found that thiazides reduced cardiovascular endpoints in comparison to placebo in older people, particularly regarding the risk of stroke, and that benefit was clinically relevant compared to risk.

For comparing thiazides to other antihypertensive drugs, the available evidence is more limited for our age group of interest. Data comparing thiazides with calcium channel blockers are available from three trials, with conflicting results. In one randomized controlled trial (ACCOMPLISH) the combination of an ACE inhibitor with hydrochlorothiazide was less effective than the combination of an ACE inhibitor with the calcium channel blocker amlodipine [40]. This is in line with the recommendations of the NICE guideline to choose a calcium channel blocker as first line therapy in adults aged over 55 years [6]. In contrast, two trials showed no significant difference between thiazides and calcium channel blockers [24, 43]. Based on this evidence, a general recommendation that thiazides are less effective than calcium channel blockers in older people could not be developed, but a specific recommendation for the combined treatment with benazepril and hydrochlorothiazide based on the evidence from ACCOMPLISH was made. This recommendation is currently discussed by the editorial team.

Data comparing thiazides with β-blockers were available from the MRC-O trial [44]. In an analysis comparing the two treatment groups, patients with hydrochlorothiazide had fewer coronary events. Studies comparing thiazides with ACE inhibitors in the treatment of hypertension for older patients could not be identified.

The included studies reported on different types of thiazides and we did not find any evidence that certain types of thiazides may be safer for older people. Beneficial effects were not restricted to any particular type of thiazide.

In the studies included in our review results were mixed regarding any association with adverse events, except for the increased risk of gout [32, 35, 44]. In the absence of studies showing clinically relevant outcomes of adverse effects in the age group ≥65 years, and taking into consideration the evidence on potential benefits, we did not find any justification for the development of a recommendation to discontinue thiazides in general for the management of hypertension in older people.

Aside from the risks of thiazide treatment, a potential protective effect of thiazides on the risk of fractures, due to the reduction of urinary calcium excretion, has been hypothesised. Two observational studies in our SR addressed this topic but showed conflicting results [42, 56]. In two other observational studies including older adults, thiazide users did not have a lower risk of hip fracture [60, 61], while another observational study showed a protective effect for long-term use, and particularly for high dose thiazide use [62]. Thus, evidence is not convincing to recommend thiazides for fracture prevention in older adults with hypertension.

Use of thiazides was associated with increased risk of gout in one observational study, and in two randomized controlled trials the incidence of gout was higher in the diuretic group [32, 35, 44]. Thiazides are listed on the STOPP list for patients with a history of gout and should be carefully considered in patients with gout according to the American College of Rheumatology Guidelines for Management of Gout [9, 59]. The observational study found no significant relationship with gout at lower doses of thiazides, although the confidence interval was wide. Furthermore, in a SR of interest on morbidity and mortality in the management of hypertension with different drugs, low doses of thiazides (25 mg/d HCT equivalent) were superior in reducing death and coronary artery disease than high doses [58], although this systematic review did not focus on older people. Considering this evidence as a whole, we developed a recommendation to adopt low dose thiazide treatment in general. This recommendation was discussed and approved by the editorial team.

A third recommendation was developed based on the evidence from the EWPHE trial where hydrochlorothiazide/triamterene demonstrated no benefit in the age group of 80 years and over. Evidence on the effectiveness of other types of thiazides in very old people was lacking, so this recommendation was restricted to this specific treatment. This recommendation is currently discussed by the editorial team and a final decision has not been reached.

## Discussion

Our systematic review investigated the benefits and risks of treatment with thiazides for the management of hypertension in older people. This systematic review was one of a series of systematic reviews on commonly used drugs in older people and aimed to identify evidence to develop recommendations relating to inappropriate use and discontinuation of these medications in older adults. Overall, the evidence suggests a benefit of thiazide treatment for the management of hypertension in older people for clinically relevant outcomes such as cardiovascular mortality and morbidity, especially stroke.

These results are in the line with the results seen in younger populations, where thiazides appear to reduce mortality, stroke, cardiovascular events and coronary heart disease [63]. Except for a reduction of mortality which was less clear in our age group, we found similar results. Despite the fact that thiazides are among the drugs that often cause hospitalisation [8, 64], this outcome was not found in the present study. We found an increased risk for gout but mixed results for other adverse events [32, 35, 44]. Frequent adverse events of thiazides include electrolyte imbalances, but our systematic review focused on clinical outcomes and we did not include studies reporting about abnormal laboratory measurements.

Based on the evidence identified, three recommendations on potential discontinuation or lowering of the dose of thiazides were developed. Although some evidence for risks of thiazide treatment was also identified, the research team considered that a general recommendation for the discontinuation of thiazide treatment in older adults would be inappropriate.

Despite the currently ongoing discussion that chlorthalidone may be superior to hydrochlorothiazide in preventing cardiovascular events we found no convincing evidence that a certain thiazide may be more beneficial in the treatment of hypertension [65]. Some studies suggest that chlorthalidone shows more benefit compared to hydrochlorothiazide [66, 67]. In contrast, we included a retrospective cohort study showing no significant difference regarding the efficacy of both drugs, but in this study chlorthalidone was associated with more adverse events in older patients. This effect, though, was mainly present for high doses of chlorthalidone [29].

The decision regarding the most beneficial antihypertensive drug class for older people is compounded by comorbidities and interactions from other drugs that need to be taken into account. None of the included studies focused on frail older people or people with multimorbidity. Furthermore, information on the functional status, cognitive status, polypharmacy or multimorbidity was scarce to missing. Polypharmacy is very common among older people, with one third of adults aged 65 or older taking 5 drugs or more per day [68, 69]. Polypharmacy increases the risk of adverse events due to interactions and may not be appropriate for all patients. Polypharmacy was not assessed in any of our included studies. The HYVET trial included very old patients aged 80 years and older, but participants were relatively healthy and fit for their age group [37]. To draw more valid conclusions in old, frail hypertensive patients with multimorbidity, research focused on this patient group is needed.

Our study has limitations. First, we found sufficient evidence for thiazides in comparison to placebo, but data on comparisons against other specific kinds of antihypertensive drugs are limited. A further database search for interventional and observational studies could have provided further data on this topic. However, our focus was not to review the evidence on thiazides in comparison to other antihypertensive drugs but to identify general risks and benefits of its treatment in older patients. Second, we did not develop any recommendations in favor of thiazides, because our recommendations are intended to be used to reduce inappropriate medication use. Furthermore, our language criteria for inclusion could have limited the number of included studies but we covered a wide range of languages.

Our recommendations will be integrated in an electronic decision support tool aimed to reduce polypharmacy in older patients. The tool is designed for general practitioners (GPs) to support them to optimize drug treatment of their patients. The patient data is entered into an electronic reporting form and the tool provides patient specific recommendations for the GP which drugs could or should be discontinued, switched to a more appropriate drug or be reduced in its dosing. GPs are trained on the use of the support tool. However, the tool does not aim at substituting the clinical judgements. Thus, decisions on the prescription or de-prescription of thiazides should be made taking the symptoms and individual characteristics of each patient into account, including any other antihypertensive medications the patient may be taking, and involving the older person in the decision-making process. This tool is currently being used and evaluated in a multicentre randomised controlled trial with 3900 patients [11].

## Conclusions

Low dose treatment with thiazide or thiazide like diuretics in adults aged 65 and older is in general appropriate. Patients with a history of gout should avoid thiazide use. At present, evidence as to whether other antihypertensives are more effective or safer is too scarce to draw definite conclusions. Patients with multimorbidity, polypharmacy and very elderly patients are underrepresented in clinical trials. The benefits for these patient groups are less clear.

## Additional files

Additional file 1: Search strings. (DOCX 15 kb)

Additional file 2: Table S1. Summary of study characteristics. (DOCX 33 kb)

Additional file 3: Table S2. Characteristics of participants in included studies. (DOCX 23 kb)

Additional file 4: Table S3. Summary of study findings. (DOCX 51 kb)

Additional file 5: Table S4. Recommendations developed for thiazide use in adults aged ≥65 years. (DOCX 15 kb)

## Abbreviations

ACE: Angiotensin converting enzyme
AMSTAR: Assessment of multiple systematic reviews
CASP: Critical Appraisal Skills Programme
CHD: Coronary heart disease
CVD: Cardiovascular disease
DARE: Database of Abstracts or Reviews of Effects
ESH/ESC: European Society of Hypertension/European Society of Cardiology
GRADE: Grading of Recommendations Assessment, Development and Evaluation
HSCS: Hypertension-Stroke Cooperative Study Group
HTA: Health Technology Assessment
HYVET: Hypertension in the Very Elderly Trial
IPA: International Pharmaceutical Abstracts
IS: Interventional study
JNC-7: Seventh report of the Joint National Committee
JNC-8: Eighth Joint National Committee guidelines
MI: Myocardial infarction
MRCO-O: Medical Research Council-Old
NICE: National Institute for Health and Care Excellence
OS: Observational study
PICOS: Population, intervention, comparison, outcomes and study design
PRIMA-eDS: Polypharmacy in chronic diseases: Reduction of Inappropriate Medication and Adverse drug events in elderly populations by electronic Decision Support
PRISMA: Preferred Reporting Items for Systemtaic Reviews and Meta-Analyses
RCT: Randomized controlled trial
SAE: Serious adverse event
SHEP: Systolic Hypertension in the Elderly Program
WHO: World Health Organization
