# Supplementary material for: Thiazides in the management of hypertension in older adults – a systematic review
Source: BMC Geriatr. 2017 Oct 16;17(Suppl 1):228. doi: 10.1186/s12877-017-0576-3 (PMC5647553; doi:10.1186/s12877-017-0576-3)
Supplement: Supplementary file 1 — Search strings. (DOCX 15 kb) [file 12877_2017_576_MOESM1_ESM.docx]

**Appendix I:** Search strings

| **Searches** |
| --- |
| Population:  geriatrics.mp. or exp geriatrics/ |
| geriatric patient.mp. |
| geriatric*.mp. |
| (elder$ or geriatric$).ab,ti. |
| elder*.mp. |
| frail elderly.mp. or exp frail elderly/ |
| aged.mp. or exp Aged/ |
| old*.mp. |
| old* adult*.mp. |
| old* people*.mp. |
| >65.mp. |
| over 65.mp. |
| Condition: |
| hypertension.mp. or exp hypertension / |
| high blood pressure |
| Drug: |
| Thiazides.mp. or exp Thiazides**/** |
| bendroflumethiazide.mp. |
| hydroflumethiazide.mp. |
| hydrochlorothiazide.mp. |
| chlorothiazide.mp. |
| polythiazide.mp. |
| trichlormethiazide.mp. |
| cyclopenthiazide.mp. |
| methyclothiazide.mp. |
| cyclothiazide.mp. |
| mebutizide.mp. |
| thiazide-like diuretic* |
| quinethazone |
| clopamide |
| chlortalidone |
| chlorthalidone |
| mefruside |
| clofenamide |
| metolazone |
| meticrane |
| xipamide |
| indapamide |
| clorexolone |
| fenquizone |
| Outcome: |
| mortality.mp. or exp mortality/ |
| quality of life.mp. or exp quality of life/ |
| QOL.mp |
| cardiovascular event.mp |
| myocardial infarction.mp |
| stroke.mp |
| hospitalization.mp. or exp hospitalization/ |
| hospitalisation.mp. or exp hospitalisation/ |
| life expectancy.mp |
| cognitive impairment.mp |
| cognitive status.mp. |
| functional status.mp. |
| functional impairment.mp. |
| renal failure.mp |
| renal insufficiency.mp. or exp renal insufficiency/ |
| adverse drug event.mp |
| adverse effects.mp. or exp adverse effects/ |
| drug toxicity.mp. or exp drug toxicity/ |
| safety.mp |
| patient safety.mp. or exp patient safety/ |
| falls.mp. |
| delirium.mp. or exp delirium/ |
| Study type: |
| (systematic review.ti. or meta-analysis.pt. or meta-analysis.ti. or systematic literature review.ti. or (systematic review.ti,ab. and review.pt.) or consensus development conference.pt. or practice guideline.pt. or cochrane database of systematic reviews.jn. or acp journal club.jn. or health technology assessment winchester england.jn. or evidence report technology assessment summary.jn. or drug class reviews.ti.) or (clinical guideline.tw and management.tw) or ((evidence based.ti. or evidence-based medicine.sh. or best practice*.ti. or evidence synthesis.ti,ab.) and (review.pt. or diseases category.mp. or behaviour.sh. and behavior mechanisms.mp. or therapeutics.sh. or evaluation studies.pt. or validation studies.pt. or guideline.pt. or pmcbook.mp.)) or ((systematic.tw or systematically.tw or critical.ti,ab. or (study selection.tw.) or (predetermined.tw or inclusion.tw and criteri*.tw) or exclusion criteri*.tw. or main outcome measures.tw. or standard of care.tw. or standards of care.tw.) and (survey.ti,ab. or surveys.ti,ab. or overview*.tw. or review.ti,ab. or reviews.ti,ab. or search*.tw. or handsearch.tw. or analysis.ti,ab. or critique.ti,ab. or appraisal.tw. or (reduction.tw. and (risk.sh. or risk.tw.) and (death.mp or recurrence.mp))) and (literature.ti,ab. or articles.ti,ab. or publications.ti,ab. or publication.ti,ab. or bibliography.ti,ab. or bibliographies.ti,ab. or published.ti,ab. or unpublished.tw. or citation.tw. or citations.tw. or database.ti,ab. or internet.ti,ab. or textbooks.ti,ab. or references.tw. or scales.tw. or papers.tw. or datasets.tw. or trials.ti,ab. or meta-analy*.tw. or (clinical.ti,ab. and studies.ti,ab.) or treatment outcome.sh. or treatment outcome.tw. or pmcbook.mp.)) not (letter.pt. or newspaper article.pt. or comment.pt.) |
|  |
