# Supplementary material for: Thiazides in the management of hypertension in older adults – a systematic review
Source: BMC Geriatr. 2017 Oct 16;17(Suppl 1):228. doi: 10.1186/s12877-017-0576-3 (PMC5647553; doi:10.1186/s12877-017-0576-3)
Supplement: Supplementary file 2 — Summary of study characteristics. (DOCX 33 kb) [file 12877_2017_576_MOESM2_ESM.docx]

**Additional file 2: Table S1 Summary of study characteristics**

| **Authors and publication year** | **Type of study** | **Aim** | **Intervention/Exposure** | **Sample size and information about the amount of older participants** | **Follow-up** | **Outcomes and measurement tools if applicable** |
| --- | --- | --- | --- | --- | --- | --- |
| **ACCOMPLISH**  Jamerson et al. 2008 | Double blind RCT | To test if the combination of a ACE inhibitor plus a calcium channel blocker would be more effective than an ACE inhibitor plus HCT in reducing cardiovascular events in high-risk patients with hypertension | Benazepril/amlodipine 20mg/5mg daily (maximum dose 40mg/10mg daily) vs.  benazepril/hydrochlorothiazide daily 20mg /12.5mg (maximum dose 40mg/25mg daily) | N = 11506  IG (Benazepril + amlodipine): n = 5744  CG (Benazepril + HCT): n = 5762  Mean age 68.4  Subgroup for ≥65 and ≥75 years | Mean 36 months | Composite of cardiovascular event (nonfatal MI, stroke, hospitalization for unstable angina, coronary revascularization, resuscitation after sudden cardiac arrest) and death from cardiovascular causes (death from MI, stroke, coronary intervention, CHF or other) |
| **ALLHAT 2002** | Double-blind, active controlled RCT | To determine whether treatment with a calcium channel blocker or an angiotensin-converting enzyme inhibitor lowers the incidence of CHD or other CVD events vs. treatment with a diuretic. | 2.5mg amlodipine (maximum dose 10mg/d)  vs. lisinopril 10mg (maximum dose 40mg/d)  vs.  12.5 mg chlorthalidone (maximum dose 25 mg/d)  At step 2 atenolol or reserpine or clinidine could be added  At step 3 hydralazine could be added | N = 33357 patients  N = 9048 amlodipine  N = 9054 lisinopril  N = 15255 chlorthalidone  Mean age 66.9 years  Subgroup for ≥ 65 years | Mean 4.9 years | Combined fatal CHD or nonfatal myocardial infarction |
| **ALLHAT 2000** | Double-blind, active controlled RCT | To determine whether newer antihypertensive agents, including doxazosin, an β-blocker, differ from chlorthalidone (12.5mg/d ) with respect to coronary heart disease and other cardiovascular disease events in hypertensive patients at high risk of coronary heart disease | Doxazosin 2 mg/d (maximum dose 8 mg/d)  vs.  12.5 mg chlorthalidone (maximum dose 25 mg/d)  At step 2 atenolol or reserpine or clinidine could be added  At step 3 hydralazine could be added | N = 24316 patients  CG (chlorthalidone) n = 15255 Patients  IG (doxazosin) n = 9061 Patients  Mean age  CG:66.9 +/-7.7 years  IG : 66.8 +/- 7.7 years  Estimated 60% >65years  Subgroup for ≥ 65 years | Mean 3.3 years | Combined occurrence of fatal CHD or nonfatal myocardial infarction  All-cause mortality, combined CHD, stroke, combined CVD |
| **ALLHAT trial** 2003  *Includes additional 9232 participant years and 939 CVD events* | double-blind, active controlled RCT | Same as ALLHAT 2000 | Same as ALLHAT 2000 | Same as ALLHAT 2000 | Mean 3.2 years | Same as ALLHAT 2000 |
| **Barzilay 2004** | Secondary analysis of ALLHAT | To study the effects of doxazosin and chlorthalidone on cardiovascular disease in years with hypertension and glucose disorders | Same as ALLHAT 2000 | Analysis for subgroups of patients with new glucose disorder (n = 1690)  Known diabetes (n = 8749)  And no glucose disorder (n = 13877) | Mean 3.09-3.25 years | Same as ALLHAT 2000 |
| **Chalmers** et al. 2000 | Double-blind RCT | To determine the long-term efficacy and safety of a fixed combination of perindopril 2mg/indapamide 0.625 mg as first line treatment in elderly patients. | Perindopril/indapamide 2mg/0.625mg daily (IG) vs. placebo (CG) | N = 383 patients  IG n = 193 patients  CG n = 190 patients | 60 weeks | Normalization of BP,  adverse events |
| **Dhalla** et al. 2013 | Propensity score–matched observational cohort study .retrospective population-based cohort study | To compare the effectiveness and safety of chlorthalidone and hydrochlorothiazide in older adults | Chlorthalidone 2.5, 25 or 50mg/d  compared to hydrochlorothiazide 12.5, 25 or 50 mg/d | N = 29873  chlorthalidone n = 10384  HCT n = 19489  Mean age 73 years (all aged ≥66 years) | Median 255 days in the chlorthalidone group and 398 days in the hydrochlorothiazide group | Composite of death or hospitalization with acute myocardial infarction, heart failure, or ischemic stroke  hospitalization for hypokalemia or hyponatremia |
| **EWPHE trial Amery** et al. 1985 | Double blind RCT | To assess effects of antihypertensive drug therapy with hydrochlorothiazide+triamteren on morbidity and mortality in patients over the age of 60 | Hydrochlorothiazide+triamteren 25/50mg daily (IG) vs. placebo  Dose could be increased to hydrochlorothiazide+triamteren 50/100mg + Metoprolol 500mg daily | n=840 Patients  IG (HCT) n= 416  CG (placebo) n= 424  Mean age 72 years  >80 years n=155 | 56 months mean | Primary outcome: all-cause mortality  Secondary outcomes: Cardiovascular mortality, cardiac mortality |
| **Amery** et al. 1985 | Secondary analysis of EWPHE | To investigate the relation of cardiovascular mortality and treated BP according to age, sex, BP and previous cardiovascular disease | Same as EWPHE (Amery et al. 1985) | Same as EWPHE (Amery et al. 1985) | Not stated | Cardiovascular mortality/cardiovascular study terminating events in relation to age, sex and previous cardiovascular mortality |
| **Fletcher** 1991 | Secondary analysis of EWPHE | To assess adverse treatment effects in elderly hypertensive patients randomly treated with hydrochlorothiazide+triamteren or placebo | Same as EWPHE (Amery et al. 1985) | Same as EWPHE (Amery et al. 1985) | Not stated | Adverse effects |
| **Staessen** et al. 1989  **Staessen** et al. 1991 | Secondary analysis of EWPHE | To investigate the relation between mortality and treated blood pressure with hydrochlorothiazide + triamteren | Same as EWPHE (Amery et al. 1985) | N = 691 patients  IG = 339  CG = 352  mean age 71.5 years | 9 month | Mortality  fall in diastolic blood pressure  changes in body weight and haemoglobin |
| **Staessen** 1991(b) | Secondary analysis of EWPHE | To determine the prognostic significance of serum uric acid in the EWPHE trial | Same as EWPHE (Amery et al. 1985) | N = 822 included in analysis  Mean age 72 years | 5 years | Uric acid levels  Incidence of gouty arthritis |
| **Gurwitz** et al. 1997 | Retrospective cohort study | To determine the risk for the initiation of treatment for gout for patients initiated on antihypertensive agents including thiazide diuretics | Thiazides | N = 9249  All patients > 65 years | 12-24 months | Rates of initiation of anti-gout therapy in relation to anti- hypertensive exposure |
| **HSCS** 1974 | Double blind RCT | To test if antihypertensive therapy for stroke survivors would alter the stroke recurrence rate | Methylclothiazide 5mg combined with deserpidine 0.5mg daily (maximum dose methylclothiazide 10mg combined with deserpidine 1mg daily) vs.  placebo | N = 452  Mean age 59 years,  Subgroup for > 70 years (n = 44 ) | 27.4 months for patients who reached an endpoint  38.6 for those without | Stroke recurrence  Cardiovascular endpoints (e.g CCF, MI, sudden death, pulmonary embolus) |
| **HYVET** Beckett et al. 2008 | Double-blind RCT | To examine the relative benefits and risks of antihypertensive treatment in patients 80 years of age or older | Indapamide 1.5 mg daily  perindopril could be added up to 4mg daily vs.  placebo | N = 3845 patients  IG: 1933 patients  CG: 1912 patients  Mean age 83.6 years | Median 1.8 years | Fatal or nonfatal stroke  Death from any cause, death from cardiovascular causes, death from cardiac causes, and death from stroke |
| **HYVET-COG** Peters et al.2008 | Substudy of HYVET | To assess the risks and benefits including cognitive function of treatment of hypertension in elderly patients | Same as HYVET (Beckett et al. 2008) | n=3336  IG: 1023  CG: 994 | Mean 2.2. years | Incidence of dementia |
| **HYVET** pilot Bulpitt et al. 2003 | RCT | To test feasibility, the safety of the active treatment and obtain a rough estimate of any treatment effects | Bendroflumethiazide 2.5 mg/d (maxiumum dose 5mg/d + diltiazem 24mg/d) or  lisinopril 2.5 mg/d (maxiumum dose 5mg/d + dilitazem 24mg/d) vs.  no treatment | N = 1283 patients  Diuretic n = 426  ACE-inhibitor n = 431 placebo n = 426 patients  Mean age 83,8 years | 13 months | Stroke events, total mortality and cardiovascular, cardiac and stroke mortality |
| **Kuramoto** et al. 1981 | Prospective clinical trial | To assess the effectiveness of antihypertensive treatment in the elderly with mild hypertension in decreasing the cerebro-vascular and cardiac complications | Trichlormethiazide 1-4mg/daily (reserpine 0.3mg, methyldopa up to 500mg/d and hydralazine up to 100mg/d could be added) vs. placebo | N = 100  IG = 44 patients  CG = 47 patients  Mean age 74.9 years | 48 months | Cerebrovascular and cardiac complications |
| **LaCroix** 1990 | Prospective case-control study | To study the effect of thiazide diuretic agents on the incidence of hip fracture | Any thiazide | N = 9518  Age: 65 or older  Mean age: users 74.0 years;  non-users: 74.3 years | 48 months | Incidence of hip fracture |
| **MRC-O** trial 1992 | Single blind RCT | To establish whether treatment with diuretic or β-blocker in hypertensive older adults reduces risk of stroke, coronary heart disease and death | 25mg/d hydrochlorothiazide + amiloride 2,5 mg/d (maximum dose 50mg/5mg daily + nifedipine up to 20mg/d) or atenolol 50mg/d (maximum dose 100mg/d + nifedipine up to 20mg/d) vs. placebo | n = 4396  Diuretic: n = 1081  ß-blocker: n = 1102  Placebo: n = 2213  Mean age ~70.3 years | Average follow up of 5.8 years | Stroke, coronary events, death |
| Bird et al. 1990 | Substudy of MRC-O | To test the effect upon cognitive function by antihypertensive treatment with thiazides+triamteren | 25mg/d hydrochlorothiazide + amiloride 2.5 mg/d or atenolol 50mg/d vs. placebo | N = 2401  mean age 70.3 years (SD 2.7) | 9 months | Cognitive performance, depression |
| **SHELL**  Malacco et al. 2003 | RCT open design | To compare the effect of lacidipine and chlorthalidone on cardiovascular outcome in elderly patients with isolated systolic hypertension | Chlorothalidone 12.5mg/day (maximum dose 25mg/d + ACE inh.) vs. lacidipine 4mg/day (maximum dose 6 mg/d + ACE inh.) | N = 1882 patients  IG (chlorthalidone)  N = 940 patients  CG (lacidipine)  N = 942 patients  Mean age  IG = 72.4 +/- 7.6 years  CG = 72.3 +/- 7.5 years | 5 years | Cardiovascular outcome  Blood pressure  TIA/ All-cause mortality |
| **SHEP-Pilot study**  Hulley et al.1985  Perry et al. 1986  Perry et al. 1989 | Double-blind RCT | To test feasibility of a RCT in participants aged 60 years and older with isolated systolic hypertension treated with chlorthalidone | Chlorthalidone 25mg/day  (maximum dose chlorthalidone 50mg/d + randomization to hydralazine, reserpine, metoprolol or placebo) vs, placebo | N = 551  IG (chlorthalidone)  N = 443  CG (placebo)  N = 108  mean age 72.1 years | average 34 months | Feasibility, stroke, all-cause mortality, cardiovascular events, adverse effects, BP |
| **SHEP** trial  SHEP Group 1991  Hawkins 1993 | Double-blind RCT | To assess the ability of antihypertensive drug treatment with chlorthalidone to reduce risk of stroke in older patients with isolated systolic hypertension | Chlorthalidone 12.5 mg/d  dose 2: (maximum dose 25 mg/d chlorthalidone  + atenolol 25 mg/d  or reserpine 0.05mg/d) vs.  placebo | N = 4736  IG n = 2365  CG n = 2371  mean age 71.6 years | 70 months  average 4.5 years | Primary outcome: nonfatal and fatal stroke  Secondary outcome: cardiovascular and coronary morbidity and mortality, all-cause mortality, quality of life |
| Perry et al. 2000 | Secondary analysis of the SHEP trial | To investigate antihypertensive the effect of drug treatment with chlorthalidone on incidence of stroke by type and subtype | Same as SHEP (SHEP group 1991) | Same as SHEP (SHEP group 1991) | Same as SHEP (SHEP group 1991) | Different types of stroke  stroke mortality rates  BP associated with stroke |
| Kostis et al. 1997 | Secondary analysis of the SHEP trial | To assess the effect of diuretic -based antihypertensive treatment on the occurrence of heart failure in older persons with isolated systolic hypertension | Same as SHEP (SHEP group 1991) | Same as SHEP (SHEP group 1991) | Same as SHEP (SHEP group 1991) | Heart failure |
| Curb et al. 1996 | Secondary analysis of the SHEP trial | To assess the effect antihypertensive treatment with chlorthalidone on major cardiovascular disease in older, non-insulin-dependent diabetic persons with ISH compared to non-diabetics | Same as SHEP (SHEP group 1991) | Same as SHEP (SHEP group 1991)  Non-insulin-dependent diabetics: 583  Non-diabetics: 4149  (4 patients not classifiable) | Same as SHEP (SHEP group 1991) | Primary outcome: nonfatal and fatal stroke  Secondary outcome: nonfatal MI+fatal CHD  major cardiovascular morbidity and mortality |
| Savage et al. 1991 | Secondary analysis of the SHEP trial | To assess the impact of treatment with chlorthalidone in older patients with isolated systolic hypertension on levels of glucose, lipid, uric acid, potassium, creatinine and new onset diabetes | Same as SHEP (SHEP group 1991) | Same as SHEP (SHEP group 1991) | Same as SHEP (SHEP group 1991) | Development of diabetes,  effects on other cardiovascular disease risk factor levels |
| Somes et al.1999 | Secondary analysis of the SHEP trial | To assess the role of treated DBP (with chlorthalidone) in stroke, CHD, CVD in patients with isolated systolic hypertension | Same as SHEP (SHEP group 1991) | Same as SHEP (SHEP group 1991)  Subgroup for ≥80 years | Same as SHEP (SHEP group 1991) | First major CVD event (stroke, TIA, MI, heart failure, coronary artery bypass surgery, angioplasty, aneurysm, endarterioectomy, sudden death, rapid cardiac death)  fatal and nonfatal stroke  fatal and nonfatal coronary disease |
| **Weiland** et al. 1997 | Case-control study | To establish the protective effect of thiazide diuretics against the risk of hip fractures among old women treated for hypertension | Any thiazide | n=725  Cases (hip fracture) n=311  Controls (no hip fracture) n=414  Mean age ~73 years | 7 years | Risk of hip fracture |
| Note: ACE: Angiotensin converting enzyme, BP: Blood pressure, CG: Control group; CHD: Coronary heart disease, CHF: Congestive heart failure, CVD: Cardiovascular disease; HCT: hydrochlorothiazide; IG: Intervention group; MI: Myocardial infarction; RCT: Randomized controlled trial, TIA: transient ischaemic attack | | | | | | |
