# Supplementary material for: Thiazides in the management of hypertension in older adults – a systematic review
Source: BMC Geriatr. 2017 Oct 16;17(Suppl 1):228. doi: 10.1186/s12877-017-0576-3 (PMC5647553; doi:10.1186/s12877-017-0576-3)
Supplement: Supplementary file 3 — Characteristics of participants in included studies. (DOCX 23 kb) [file 12877_2017_576_MOESM3_ESM.docx]

**Additional file 3: Table S2: Characteristics of participants in included studies**

| **Authors and publication year** | **Setting / country / ethnicity** | **Male sex** | **Age** | **Stage of disease** | **Comorbidity** | **Polypharmacy** | **Functional status / Frailty level** | **Cognitive status** |
| --- | --- | --- | --- | --- | --- | --- | --- | --- |
| **ACCOMPLISH**  Jamerson et al. 2008 | Multicentre trial in US and North Europe | 60.5% | Mean 68.4 years  IG (benazepril+amlodipine): 68.4 (SD 6.86)  CG (benazepril+HCT): 68.3 (SD 6.86)  Subgroup analyses for ≥65 and for ≥75 years | Not described | Previous MI (IG/CG) 23.3/23.8%  previous stroke 13.3/12.8%  previous hospitalisation for unstable angina 11.4/11.6%  Diabetes mellitus 60.6/60.2%  renal disease 6.1%  left ventricular hypertrophy 13.3/13.2%  Atrial fibrillation 6.5/7.0% | ≥3 antihypertensive drugs 37.4/39.4% | Not described | Not described |
| **ALLHAT 2002** | 623 centres in the United States, Canada, Puerto Rico, and the US Virgin Islands | Amlodipine: 52.7%  lisinopril: 53.8%  chlorthalidone: 53% | Amlodipine: mean age 66.9 years (SD:7.7)  lisinopril: mean age 66.9 years (SD: 7.7)  chlorthalidone: mean age 66.9 years (SD: 7.7)  Subgroup analysis for ≥65 years | Inclusion criteria stage 1 or 2 hypertension | Atherosclerotic CVD:  Amlodipine 51.0%  Lisinopril 51.7% chlorthalidone 51.8%  History of CHD:  Amlodipine 24.5%  Lisinopril 25.3% chlorthalidone 26.0%  Type 2 diabetes:  Amlodipine 36.7%  Lisinopril 35.5% chlorthalidone 36.2% | Not described | Not described | Not described |
| **ALLHAT 2000** | 625 centres in US and Canada | IG (doxazosin) = 53.6 %  CG (chlorthalidoen)= 53% | IG = 67 years (SD 8)  CG = 67 years (SD8)  Subgroup analysis for ≥65 years | Same as ALLHAT 2002 | Atherosclerotic cardiovascular disease IG: 45.8%/CG: 45.9%  Type 2 diabetes IG: 36.2/CG: 35.5%  LVH by ECG within 2 years IG: 16.2%/CG: 16.3% | Not described | Not described | Not described |
| **ALLHAT** 2003 | 623 centers in the United States, Canada, Puerto Rico, and the US Virgin Islands | IG = 53.6 %  CG = 53% | IG: 66.8 +/- 7.7  CG (CHT): 66.9 +/-7.7 years  years  Subgroup analysis for ≥65 years | Same as ALLHAT 2002 | Atherosclerotic cardiovascular disease IG: 45.8%/CG: 45.9%  Type 2 diabetes IG:36.2/CG:35.5%  LVH by ECG within 2 years IG: 16.2%/CG: 16.3% | Not described | Not described | Not described |
| **Barzilay** et al. 2004 | Same as ALLHAT 2002 | Same as ALLHAT 2002 | Same as ALLHAT 2002 | Same as ALLHAT 2002 | See above analysis according to glucose disorder, known diabetes or no glucose disorder | Not described | Not described | Not described |
| **Cauley** et al. 1993 | 4 clinical centres in US | 0% | 100% > 65 years  Age according to thiazide use:  Never =71.4 ± 5.2 years  Past= 71.4 ± 5.3 years  Current and for 10 or Fewer Years = 72.4 ± 5.5 years  Current and for More than 10 Years =  72.7 ± 5.6 years | Not described | Surgical menopause (11-14%) | Not described | Functional level assessed, but results not reported | Not described |
| **Chalmers** et al. 2000 | France and Australia | IG: 41%  CG: 40% | Aged 65-85 years  Mean age 72 years  34% >75years | Not described | Not described | Not described | Not described | Not described |
| **EWPHE** Amery et al. 1985 | Multi-centre trial in Europe | 29% men overall. Placebo 29.4, Intervention 30.1 | 72 +/- 8 years  in both groups  (IG= 71.4 years  CG = 71.7years) | Not described | Not described | Not described | Not described | Not described |
| Amery et al. 1985 | Same as EWPHE (Amery 1985) | Same as EWPHE (Amery 1985) | Same as EWPHE (Amery 1985) | Same as EWPHE (Amery 1985) | Same as EWPHE (Amery 1985) | Same as EWPHE (Amery 1985) | Same as EWPHE (Amery 1985) | Same as EWPHE (Amery 1985) |
| Fletcher et al. 1991 | Same as EWPHE (Amery 1985) | Same as EWPHE (Amery 1985) | Same as EWPHE (Amery 1985) | Same as EWPHE (Amery 1985) | Same as EWPHE (Amery 1985) | Same as EWPHE (Amery 1985) | Same as EWPHE (Amery 1985) | Same as EWPHE (Amery 1985) |
| Staessen et al. 1989  Staessen et al. 1991 | Same as EWPHE (Amery 1985) | Same as EWPHE (Amery 1985) | Same as EWPHE (Amery 1985) | Same as EWPHE (Amery 1985) | Same as EWPHE (Amery 1985) | Same as EWPHE (Amery 1985) | Same as EWPHE (Amery 1985) | Same as EWPHE (Amery 1985) |
| Staessen 1991 (b) | Same as EWPHE (Amery 1985) | Same as EWPHE (Amery 1985) | Same as EWPHE (Amery 1985) | Same as EWPHE (Amery 1985) | Same as EWPHE (Amery 1985) | Same as EWPHE (Amery 1985) | Same as EWPHE (Amery 1985) | Same as EWPHE (Amery 1985) |
| **Gurwitz** et al. 1997 | New Jersey | 23% | All ≥65 years Age 65-74: 45%, age 75-84: 35%, age ≥85: 20% | Not described | Not described | Not described | Not described | Not described |
| **HSCS** 1974 | Multicenter trial in US | 60% | Mean age 59 years  75% 50-70 years 16% <50 years 9% >70 years | Not described | Not described | Not described | Not described | Not described |
| **HYVET** Beckett et al. 2008 | Western and Eastern Europe, China, Australasia, and Tunisia | G: 39.3% male  CG: 39.7% male | Mean age 83,5 (± 3.1) years (100% >80 years)  73.0% 80-84 years  22.4% 85-89 years  4.6% ≥90 years | Not described | For IG/CG  CV: 11.5/12.0%  Known Hypertension: 89.9/89.9%  History of stroke 6.7/6.9%  History of MI 3.1/3.2%  HF 2.9/2.9%  Diabetes 6.9% | Not described | Not described | Not described |
| **HYVET-COG** Peters et al.2008 | Wester/eastern Europe, China, Tunisia, southeast Asia and Australia | 39.5% | Aged 83.5 (± 3.1) years (100% >80 years) | Not described | Previous stroke:  IG: 6%  CG: 7%  Previous CVD:  IG: 11%  CG: 12% | Not described | Not described | No patients with diagnosis of dementia |
| **HYVET pilot** Bulpitt et al. 2003 | Multicentre trial in Europe | 36.4% | Overall mean age 83.8 ± 3.0 (SD) years (range 79.5–96.1 years) | Not described | Diuretic/ACE-inh./no treatment group  Previous MI 2.4/3.0/3.5%  Previous stroke 4.2/4.2/5.2% | Not described | Not described | Not described |
| **Kuramoto** et al. 1981 | Japan | 54.9% | Mean age (years)  IG (male)  74.9 (+/- 5.4)  CG (male)  75.7 (+/- 7.3)  CG (male) 76.3 (+/- 4.6)  IG (female)  77.9 (+/- 6,7)  CG (female) | Not described | Not described | Not described | All patients with “normal activity of daily living” | Not described |
| **LaCroix** 1990 | USA | 25.8% male thiazide users; 42.9% male nonusers | Mean age: Users 74.0 years / Nonusers 74.3 years (all ≥65 years)  Age groups 65-74 and >75 years | Not described | User 14.3% diabetes/non-users 12.2% diabetes | Not described | Impaired mobility: users 28.2%/non-users 24.4% | Not described |
| **MRC-O** 1992 | UK | 41.8% | Mean age approximately 70.3 years  range 65-74  46% 65-70 years  54% 70-75 years | Not described | Not described | Not described | Not described | Not described |
| Bird et al. 1990 | UK | 41% | Mean age ~70.3 years (SD 2.7) range 65-74 | Not described | Depression: present in 9.9% | Not described | Not described | 15%/36% (PALT/TMT) pathological testing |
| **SHELL**  Malacco et al. 2003 | Italy | Intervention 39.6%  Control 37.8% | IG= 72.3 +/- 7.5 years  CG= 72.4 +/- 7.6 years  19.9% ≥80 years | Not described | History of CVD (Chlorthalidone/Lacidipine) 29.2/32.1%  Diabetes 12.7/13.8% | Not described | Not described | Not described |
| **SHEP-pilot**  Hulley et al.1985  Perry et al. 1986  Perry et al. 1989 | 5 clinical centres USA | 37% | Mean age 72.1 years  61% >70 years  15% >80 years | Not described | 4.5% history of MI, 1.5.% history of stroke, 0.5% CHF during the past year, 3.3% claudicatio intemittens, 1.8% seriously depressed | Not described | Activity limitation 7% | Cognitive impairment 2% |
| **SHEP**  SHEP Group 1991  Hawkins 1993 | Multicentre trial USA | 43.2%  IG 43.7%  CG: 42.7% | Mean age 71.6 years (SD 6.7)  IG 71.5 years  CG 71.6 years | Not described | 4.9% history of MI, 1.4.% history of stroke, 10.1% history of diabetes, 11.1% depression symptoms | Not described | No limitation of activities of daily living: 94.6% | 0.4 cognitive impairment |
| Perry et al. 2000 | Same as SHEP (SHEP Group 1991) | Same as SHEP (SHEP Group 1991) | Same as SHEP (SHEP Group 1991) | Same as SHEP (SHEP Group 1991) | Same as SHEP (SHEP Group 1991) | Same as SHEP (SHEP Group 1991) | Same as SHEP (SHEP Group 1991) | Same as SHEP (SHEP Group 1991) |
| Kostis et al. 1997 | Same as SHEP (SHEP Group 1991) | Same as SHEP (SHEP Group 1991) | Same as SHEP (SHEP Group 1991) | Same as SHEP (SHEP Group 1991) | Same as SHEP (SHEP Group 1991) | Same as SHEP (SHEP Group 1991) | Same as SHEP (SHEP Group 1991) | Same as SHEP (SHEP Group 1991) |
| Curb et al. 1996 | Same as SHEP (SHEP Group 1991) | 42%  diabetics:  IG: 53%  CG: 48%  non-diabetics:  IG: 42.4%  CG: 41.9% | Diabetics:  IG: 70.2 years  CG: 70.5 years  non-diabetics: IG: 71.8 years  CG: 71.7 years | Same as SHEP (SHEP Group 1991) | Same as SHEP (SHEP Group 1991) | Same as SHEP (SHEP Group 1991) | No limitations of activities of daily living: 92.0-95.5% | Same as SHEP (SHEP Group 1991) |
| Savage et al. 1991 | Same as SHEP (SHEP Group 1991) | Same as SHEP (SHEP Group 1991) | Same as SHEP (SHEP Group 1991) | Same as SHEP (SHEP Group 1991) | Same as SHEP (SHEP Group 1991) | Same as SHEP (SHEP Group 1991) | Same as SHEP (SHEP Group 1991) | Same as SHEP (SHEP Group 1991) |
| Somes et al.1999 | Same as SHEP (SHEP Group 1991) | Same as SHEP (SHEP Group 1991) | Same as SHEP (SHEP Group 1991) | Same as SHEP (SHEP Group 1991) | Same as SHEP (SHEP Group 1991) | Same as SHEP (SHEP Group 1991) | Same as SHEP (SHEP Group 1991) | Same as SHEP (SHEP Group 1991) |
| **Weiland** et al. 1997 | Western Germany | 0% | Cases = 73.9 (± 2.9) years  Controls =73.(±3.0) years | Not described | Cardiac insufficiency cases 59.8% controls 50.9%  CHD cases 51.1% controls 52.9%  Renal insufficiency cases 7.7% controls 10.9%  Cerebral insufficiency cases 40.8% controls 23.49%  Rheumatism cases 23.5% controls 25.8% | Not described | Good/slightly impaired Cases: 88.4%  Controls: 87.2%  Several impaired:  Cases 11.6%  Controls 12.8% | Not described |
| Note: CG: Control group; CHD: Coronary heart disease, CVD: Cardiovascular disease; ECG: Electrocardiogram, HCT: Hydrochlorthiazide, IG: Intervention group; LVH: Left ventricular hypertrophy, MI: Myocardial infarction; RCT: Randomized controlled trial; SD: Standard deviation | | | | | | | | |
