# Supplementary material for: Thiazides in the management of hypertension in older adults – a systematic review
Source: BMC Geriatr. 2017 Oct 16;17(Suppl 1):228. doi: 10.1186/s12877-017-0576-3 (PMC5647553; doi:10.1186/s12877-017-0576-3)
Supplement: Supplementary file 4 — Summary of study findings. (DOCX 51 kb) [file 12877_2017_576_MOESM4_ESM.docx]

**Additional file 4: Table S3 Summary of study findings**

| **Authors and publication year** | **Outcomes** | **Thiazides**  **cases/n***  **(absolute risk %)** | **Comparator**  **cases/n***  **(absolute risk %)** | **Risk ratio^$^**  **(95% CI)** | **Reported Statistical comparison**  **(95% CI)** |
| --- | --- | --- | --- | --- | --- |
| **Mortality** | | | | | |
| **EWPHE**  Amery et al.1985  Staessen et al. 1989  Staessen et al. 1991 | All-cause mortality  Cardiovascular mortality  Mortality (at 9 month FU)  Lowest third of treated BP  Middle third of treated BP  Highest third of treated BP | HCT/triamterene:  135/416 (32.5)  67/416 (16.3)  26/120 (21.7)  13/126 (10.3)  17/106 (16.0) | Placebo:  149/424 (35.1)  93/424 (21.9)  22/112 (19.6)  20/113 (17.7)  23/105 (21.9) | 0.92 (0.76-1.12)  0.73 (0.55-0.97)  1.10 (0.67-1.83)  0.58 (0.30-1.12)  0.73 (0.42-1.29) | P = 0.41  P = 0.037  NR  P <0.05  NR |
| **HYVET**  Beckett et al. 2008 | Death from any cause  Death from cardiovascular causes | Indapamide  196/1933  (10.1) 99/1933 (5.1) | Placebo  235/1912 (12.3)  121/1912 (6.3) | 0.82 (0.69-0.99)  0.81 (0.63-1.05) | HR 0.79 (0.65-0.95)  HR 0.77 (0.60-1.01) |
| **HYVET pilot**  Bulpitt et al. 2003 | total mortality  Cardiovascular mortality  Stroke mortality | Bendroflumethiazide  30/426 (7.0)  23/426 (5.4)  6/426 (1.4) | No treatment  22/426 (5.2)  19/426 (4.5)  11/426 (2.6) | 1.36 (0.80-2.32)  1.21 (0.67-2.19)  0.55 (0.20-1.46) | HR 1.31 (0.75-2.27)  HR 1.17 (0.63-2.14)  HR 0.52 (0.19-1.42) |
| **Kuramoto** et al. 1981 | Death | Trichlormethiazide  3/38 (7.9) | Placebo  3/41 (7.3) | 1.08 (0.23-5.02) |  |
| **MRC-O** 1992  Bird 1990 | All-cause mortality  All cardiovascular deaths | HCT plus amiloride  134 events in 6,290 patient years  66 events in 6,290 patient years | Placebo  315 events in 12,735 patient years  180 events in 12,735 patient years | 0.86 (0.70-1.06)  0.74 (0.56-0.98) | NR  NR |
| **SHEP**  SHEP Group 1991  Hawkins et. al 1993  Perry et al. 2000  Curb et al. 1996 | All-cause mortality  All-cause mortality  Diabetics:  Non-diabetics: | Chlorthalidone  213/2365 (9.0)  39/283 (13.8)  173/2080 (8.3) | Placebo  242/2371 (10.2)  48/283 (17.0)  173/2080 (8.3) | 0.88 (0.74-1.05)  0.81 (0.55-1.20)  0.90 (0.74-1.09) | RR 0.87 (0.73-1.05)  RR 0.74 (0.46-1.18)  RR 0.85 (0.68-1.06) |
| **SHEP-pilot**  Hulley et al.1985  Perry et al. 1986  Perry et al. 1989 | All-cause mortality | Chlorthalidone  32/443 (7.2) | Placebo  7/108 (6.5) | 1.11 (0.51-2.46) | OR 1.12 (0.48-2.62) |
| **ALLHAT 2002**  *Subgroup ≥65 years* | All-cause mortality | Chlorthalidone  NR/8784 | Amlodipine  NR/5204  Lisinopril  NR/5185 | NR  NR | RR 1.04 (0.97-1.14)  RR 0.97 (0.89-1.05) |
| **MRC-O** 1992  Bird 1990 | All-cause mortality  All cardiovascular deaths | HCT plus amiloride  134 events in 6,290 patient years  66 events in 6,290 patient years | ß-blocker  167 events in 6,330 patient years  95 events in 6,330 patient years | 0.81 (0.64-1.01)  0.70 (0.51-0.96) | Reduction of 19% (-2%-36%), p = 0.07  NR |
| **SHELL**  Malacco et al. 2003 | All-cause mortality | Chlorthalidone  122/940 (13.0) | Lacidipine  145/942 (15.4) | 0.84 (0.67-1.05) | HR 0.81 (0.64-1.03) |
| **Stroke** | | | | | |
| **HSCS** 1974  *Subgroup >70 years* | Stroke recurrence | Deserpidine+ Methylchlothiazide  2/19 (10.5) | Placebo  9/25 (36.0) | 0.29 (0.07-1.20) | P = 0.08 |
| **HYVET**  Beckett et al. 2008 | Stroke  Fatal stroke | Indapamide  51/1933 (2.6)  27/1933 (1.40) | Placebo  69/1912 (3.6)  42/1912 (2.20) | 0.73 (0.51-1.04)  0.64 (0.39-1.03) | HR 0.70 (0.49-1.01.)  HR 0.61 (0.38-0.99) |
| **HYVET pilot**  Bulpitt et al. 2003 | Fatal stroke  Fatal and nonfatal stroke | Bendroflumethiazide  6/426 (0.9)  6/426 (0.9) | No treatment  11/426 (2.6)  18/426 (4.2) | 0.55 (0.20-1.46)  0.33 (0.13-0.83) | HR 0.52 (0.19-1.42)  HR 0.31 (0.12-0.79) |
| **MRC-O** 1992  Bird 1990 | Fatal or non-fatal stroke  Fatal stroke | HCT plus amiloride  45 events in 6,290 patient years  16 events in 6,290 patient years | Placebo  134 events in 12,735 patient years  ß-blocker  56 events in 6,330 patient years  placebo  42 events in 12,735 patient years  ß-blocker  21 events in 6,330 patient years | 0.68 (0.49-0.95)  0.81 (0.55-1.20)  0.77 (0.43-1.37)  0.77 (0.40-1.47) | NR  P = 0.33  NR  NR |
| **SHEP**  SHEP Group 1991  Hawkins et. al 1993  Perry et al. 2000  Curb et al. 1996 | Nonfatal and fatal stroke  Ischemic stroke  Hemorrhagic stroke  Unknown type of stroke  TIA  Nonfatal and fatal strokes  Diabetics:  Non-diabetics: | Chlorthalidone  103/2365 (4.4)  85/2365 (3.6)  9/2365 (0.4)  9/2365 (0.4)  62/236 (2.6)  25/283 (8.8)  78/2080 (3.8) | Placebo  159/2371 (6.7)  132/2371 (5.6)  19/2371 (0.8)  8/2371 (0.3)  82/2371 (3.5)  36/300 (12.0)  123/2069 (5.9) | 0.65 (0.51-0.83)  0.65 (0.49-0.84)  0.47 (0.22-1.05)  1.13 (0.44-2.92)  0.76 (0.55-1.05)  0.74 (0.45-1.19)  0.63 (0.48-0.83) | RR 0.64 (0.50-0.82)  RR 0.63 (0.48-0.82)  RR 0.46 (0.21-1.02)  RR 1.05 (0.40-2.73)  RR 0.74 (0.54-1.04)  RR 0.78 (0.45-1.34)  RR 0.62 (0.46-0.83) |
| **SHEP-pilot**  Hulley et al.1985  Perry et al. 1986  Perry et al. 1989 | First stroke | Chlorthalidone  11/443 (2.5) | Placebo  4/108 (4.3) | 0.67 (0.22-2.06) | OR 0.66 (0.21-2.12) |
| **ALLHAT 2002**  *Subgroup ≥65 years* | Stroke | Chlorthalidone  NR/8784 | Amlodipine  NR/5204  Lisinopril  NR/5185 | NR  NR | RR 1.08 (0.93-1.23)  RR 0.88 (0.77-0.88) |
| **SHELL**  Malacco et al. 2003 | Stroke  TIA | Chlorthalidone  38/940 (4.0)  13/940 (1.4) | Lacidipine  37/942 (3.9)  15/942 (1.6) | 1.03 (0.66-1.60)  0.87 (0.42-1.82) | HR 1.04 (0.66-1.64)  HR 0.88 (0.42-1.85)) |
| **Cardiovascular disease/Coronary heart disease** | | | | | |
| **SHEP**  SHEP Group 1991  Hawkins et. al 1993  Perry et al. 2000  Curb et al. 1996 | CHD  CVD  Major CVD events  Diabetics:  Non-diabetics:  Major CHD events  Diabetics:  Non-diabetics: | Chlorthalidone  140/2365 (5.9)  289/2365 (12.2)  57/283 (20.1)  231/2080 (11.1)  23/283 (8.1)  116/2080 (5.6) | Placebo  184/2371 (7.8)  414/2371 (17.5)  83/300 (27.7)  330/2069 (16.0)  44/300 (14.7)  139/2069 (6.7) | 0.76 (0.62-0.94)  0.70 (0.61-0.80)  0.73 (0.54-0.98)  0.70 (0.60-0.81)  0.55 (0.34-0.89)  0.83 (0.65-1.05) | RR 0.75 (0.60-0.94)  RR 0.68 (0.58-0.79)  RR 0.66 (0.46-0.94)  RR 0.66 (0.55-0.79)  RR 0.44 ( 0.25-0.77)  RR 0.81 (0.62-1.05) |
| **ALLHAT 2002**  *Subgroup ≥65 years* | Combined CHD  Combined CVD | Chlorthalidone  NR/8784  NR/8784 | Amlodipine  NR/5204  Lisinopril  NR/5185  Amlodipine  NR/5204  Lisinopril  NR/5185  Doxazosin  NR | NR  NR  NR  NR  NR | RR 0.96 (0.89-1.04)  RR 0.90 (0.83-0.97)  RR 0.95 (0.89-1.01)  RR 0.88 (0.83-0.94)  RR 0.77 (0.71-0.83) |
| **ALLHAT** 2003  *Subgroup ≥65 years* | Combined CVD | Chlorthalidone  NR/15255 | Doxazosin  NR/9061 | NR | RR 0.81 (0.76-0.88) |
| **Heart failure** | | | | | |
| **SHEP**  SHEP Group 1991  Hawkins et. al 1993  Perry et al. 2000 | Nonfatal HF  Nonfatal hospitalized HF  Fatal and non-fatal HF  Fatal and hospitalized nonfatal HF  Fatal HF | Chlorthalidone  48/2365 (2.0)  38/2365 (1.6)  55/2365 (2.3)  45/2365 (1.9)  7/2365 (0.3) | Placebo  102/2371 (4.3)  75/2371 (3.2)  105/2371 (4.4)  79/2371 (3.3)  3/2371 (0.1) | 0.47 (0.34-0.66)  0.51 (0.35-0.75)  0.53 (0.38-0.72)  0.57 (0.40-0.82)  2.34 (0.61-9.04) | RR 0.46 (0.33-0.65)  RR 0.50 (0.34-0.74)  RR 0.51 (0.37-0.71)  RR 0.57 (0.34-0.81)  P = 0.22 |
| **ALLHAT 2002**  *Subgroup ≥65 years* | Heart failure  Chronic heart failure | Chlorthalidone  NR/8784 | Amlodipine  NR/5204  Lisinopril  NR/5185  Doxazosin  NR | NR  NR  NR | RR 0.75 (0.67-0.84)  RR 0.83 (0.74-0.94)  RR 0.45 (0.41-0.56) |
| **ALLHAT** 2003  *Subgroup ≥65 years* | Heart failure | Chlorthalidone  NR/15255 | Doxazosin  NR/9061 | NR | RR 0.53 (0.46-0.61) |
| **Barzilay** et al. 2004  *Subgroup ≥65 years* | Heart failure in patients with:  New glucose disorder  Known diabetes  No glucose disorder | Chlorthalidone  NR (n~540)  NR (n~2760)  NR (n~4320) | Doxazosin  NR (n~300)  NR (n~1610)  NR (n~2620) | NR  NR  NR | RR 0.57 (0.35-0.93)  RR 0.53 (0.43-0.65)  RR 0.51 (0.42-0.62) |
| **Combined cardiovascular/cerebrovascular endpoints** | | | | | |
| **EWPHE**  Amery et al.1985 | Non-fatal cardiovascular study terminating event | HCT/triamterene:  11/416 (2.6) | Placebo:  25/424 (5.9) | 0.45 (0.22-0.90) | P = 0.0064  Reduction of -60% (-88%, -19%) |
| **HSCS** 1974  *Subgroup >70 years* | Stroke or cardiovascular recurrence  Cardiovascular endpoints | Deserpidine+ Methylchlothiazide  3/19 (15.8)  1/19 (5.3) | Placebo  12/25 (0.5)  3/25 (12.0) | 0.33 (0.11-1.00)  0.44 (0.05-3.89) | P = 0.06  P = 0.62 |
| **Kuramoto** et al. 1981 | Cerebrovascular and cardiac complications | Trichlormethiazide  4/38 (10.5) | Placebo  9/41 (22.0) | 0.48 (0.16-1.43) | P >0.05 |
| **MRC-O** 1992  Bird 1990 | Fatal or non-fatal coronary events | HCT plus amiloride  48 events in 6,290 patient years | Placebo  159 events in 12,735 patient years  ß-blocker  80 events in 6,330 patient years | 0.61 (0.44-0.84)  0.61 (0.42-0.87) | NR  P = 0.006 |
| **SHEP**  SHEP Group 1991  Hawkins et. al 1993  Perry et al. 2000  Curb et al. 1996 | Nonfatal MI or CHD death  Fatal/nonfatal stroke, nonfatal MI or CHD death  Cardiac mortality and nonfatal hospitalized HF  Cardiovascular mortality and nonfatal hospitalized HF  Nonfatal MI and fatal CHD  Diabetics:  Non-diabetics: | Chlorthalidone  104/2365 (4.4)  199/2365 (8.4)  113/2365 (4.8)  123/2365 (5.2)  18/283 (6.4)  85/2080 (4.1) | Placebo  141/2371 (6.0)  289/2371 (12.2)  162/2371(6.8)  174/2371 (7.3)  34/300 (11.3)  106/2069 (5.1) | 0.74 (0.58-0.95)  0.69 (0.58-0.82)  0.70 (0.55-0.88)  0.71 (0.57-0.89)  0.56 (0.32-0.97)  0.80 (0.60-1.05) | RR 0.73 (0.57-0.94)  RR 0.67 (0.56-0.80)  RR 0.69 (0.54-0.87)  RR 0.70 (0.55-0.87)  RR 0.46 ( 0.24-0.88)  RR 0.77 (0.57-1.05) |
| **SHEP-pilot**  Hulley et al.1985  Perry et al. 1986  Perry et al. 1989 | Hypertensive events  Atherosclerotic events | Chlorthalidone  18/443 (4.1)  26/443 (5.9) | Placebo  10/108 (9.3)  10/108 (9.3) | 0.44 (0.21-0.92)  0.63 (0.32-1.27) | NR  NR |
| **ACCOMPLISH**  **Jamerson et al. 2008**  *Subgroup ≥65 years* | Composite of cardiovascular event and death from cardiovascular causes  Age ≥65 years  Age ≥70 years | Benazepril/HCT  474/3813 (12.4)  323/2340 (13.8) | Benazepril/Amlodipine  386/3813 (10.1)  260/2363 (11.0) | 1.23 (1.08-1.39)  1.25 (1.08-1.46) | HR 1.23 (1.08-1.41)  HR 1.26 (1.08-1.49) |
| **ALLHAT 2002**  *Subgroup ≥65 years* | Non-fatal MI+fatal CHD | Chlorthalidone  NR/8784 | Amlodipine  NR/5204  Lisinopril  NR/5185 | NR  NR | RR 1.03 (0.92-1.14)  RR 0.99 (0.89-1.10) |
| **SHELL**  Malacco et al. 2003 | Composite of fatal/non-fatal stroke, sudden death, fatal/non-fatal MI, fatal/non-fatal CHF, myocardial revascularization, carotid endarterectomy | Chlorthalidone  88/940 (9.4) | Lacidipine  90/942 (9.6) | 0.98 (0.74-1.30) | HR 0.99 (0.74-1.33) |
| **New onset diabetes** | | | | | |
| **SHEP**  Savage et al. 1991 | New cases of diabetes  Year 1  Year 3 | Chlorthalidone  68/1870 (3.6)  140/1631 (8.6) | Placebo  52/1810 (2.9)  118/1578 (7.5) | 1.27 (0.89-1.81)  1.15 (0.91-1.45) | P = 0.19  P = 0.25 |
| **New onset of gout** | | | | | |
| **EWPHE**  Fletcher et al. 1991  Staessen 1991 (b) | Gout | HCT/triamteren:  7/416 (1.68) | Placebo  1/424 (0.24) | 7.13 (0.88-57.74) | P <0.05 |
| **Gurwitz** et al. 1997 | Initiation of anti-gout therapy  All patients  thiazide <25mg/day  25-49 mg/day  50+ mg/day  Initiation of anti-gout therapy | Thiazide with or without other antihypertensive medications  59 events in 4162 patient years  NR  NR  NR  Thiazide alone  22 events in 1521 patient years | No antihypertensive medication  81 events in 8927 patient years  NR  NR  NR  No antihypertensive medication  81 events in 8927 patient years | 1.56 (1.12-2.18)  1.59 (0.99-2.55) | RR 2.29 (1.55-3.37)  RR 1.78 (0.66-4.78)  RR 2.39 (1.29-4.43)  RR 2.41 (1.39-4.19)  RR 1.99 (1.21-3.26) |
| **Dementia/low cognitive performance** | | | | | |
| **HYVET-COG**  Peters et al.2008 | Cognitive decline  Alzheimer`s disease  Vascular dementia  All dementia | Indapamide  485/1687 (28.8)  78/1687 (4.6)  41/1687 (2.4)  126/1687 (7.5) | Placebo  486/1649 (29.5)  86/1649 (5.2)  43/1649 (2.6)  137/1649 (8.3) | 0.98 (0.88-1.08)  0.89 (0.66-1.20)  0.93 (0.61-1.42)  0.90 (0.71-1.13) | HR 0.93 (0.82-1.05)  HR 0.85 (0.63-1.15)  HR 0.87 (0.57-1.34)  HR 0.86 (0.67-1.09) |
| **MRC-O** 1992  Bird 1990 | Low Cognitive performance (9 mths) | HCT plus amiloride  118/556 (21.2) | Placebo  206/1116 (18.5) | 1.15 (0.94-1.41) | NR |
| **Depression** | | | | | |
| **MRC-O** 1992  Bird 1990 | Depression (9 mths) | HCT plus amiloride  57/550 (10.4) | Placebo  100/1249 (9.2) | 1.29 (0.95-1.76) | NR |
| **Fractures** | | | | | |
| **LaCroix** et al. 1990 | Hip fracture | Thiazide  NR/2566 | No thiazide  NR/6952 |  | RR 0.68 (0.49-0.94) |
| **Weiland** et al. 1997 | Risk of hip fracture  All thiazide users  Current thiazide use  Former thiazide use | Thiazide alone or combined with other agents (12 out of 225 patients had thiazide alone)  163/225 (72.3)  NR  NR | No thiazide  229/308 (74.4)  NR  NR | 0.97 (0.88,1.08) | OR 0.93 (0.65-1.34)  OR 1.11 (0.73-1.68)  OR 0.85 (0.54-1.34) |
| **Adverse events** | | | | | |
| **Chalmers** et al. 2000 | SAEs related to study medication  AEs | Perindopril/Indapamide  2/193 (1.0)    0.37 per patient year (n=138 patients) | Placebo  2/190 (1.1)  0.28 per patient year  (n=61 patients) | 0.98 (0.14-6.92)  1.32 (1.05-1.66) | NR  NR |
| **HYVET**  Beckett et al. 2008 | SAEs | Indapamide  358/1933 | Placebo  448/1912 | 0.79 (0.70-0.89) | P = 0.001 |
| **SHEP**  SHEP Group 1991  Hawkins 1993 | Troublesome symptoms  Intolerable troublesome symptoms | Chlorthalidone  2171/2365 (91.8)  665/2365 (28.1) | Placebo  2049/2371 (86.4)  493/2371 (20.8) | 1.06 (1.04-1.08)  1.35 (1.22-1.50) | NR  NR |
| **SHEP-pilot**  Hulley et al.1985  Perry et al. 1986  Perry et al. 1989 | Troublesome symptoms  Intolerable troublesome symptoms | Chlorthalidone  194/443 (44.0)  13/443 (3.0) | Placebo  52/108 (44.0)  7/108 (6.0) | 0.91 (0.73-1.14)  0.45 (0.19-1.11) | NR  NR |
| **EWPHE**  Fletcher et al. 1991  Staessen 1991 (b) | Stopped medication due to side effects or concomitant disease  Symptoms differing significantly between arms:  Dry mouth  Nasal stuffiness  Diarrhea | HCT/triamterene:  14/416 (3.4)  42% (based on approx. 266 patients)  27% (based on approx. 266 patients)  25% (based on approx. 266 patients) | Placebo  7/424 (1.7)  29% (based on approx. 270 patients)  21% (based on approx. 270 patients)  18% (based on approx. 270 patients) | 2.04 (0.83-5.0)  NR  NR  NR | NR  P <0.05  P <0.05  P <0.05  P <0.05 |
| **MRC-O** 1992 | Withdrawals due to major side effects  Reasons for withdrawal differing significantly between arms:  Impaired glucose tolerance  Gout  Skin disorders  Muscle cramp  Nausea  Dizziness | HCT (25mg daily) plus amiloride (2.5mg daily)  160 in 6,290 patient years  43 in 6,290 patient years  28 in 6,290 patient years  25 in 6,290 patient years  33 in 6,290 patient years  47 in 6,290 patient years  47 in 6,290 patient years | Placebo  82 in 12,735 patient years  34 in 12,735 patient years  1 in 12,735 patient years  14 in 12,735 patient years  1 in 12,735 patient years  14 in 12,735 patient years  15 in 12,735 patient years | 3.94 (3.02-5.15)  2.56 (1.63-4.01)  44.0 (5.99-323.4)  3.55 (1.84-6.82)  52.0 (7.11-380.2)  6.73 (3.70-12.22)  6.17 (3.45-11.03) | NR  P <0.05  P <0.05  P <0.05  P <0.05  P <0.05  P <0.05 |
| **SHELL**  Malacco et al. 2003 | Dizziness  Fatigue  Headache  Edema  Skin rash  Itching  Skeletal muscle disorders  Parasthesia  Constipation  Orthostatic hypotension  Cough | Chlorthalidone  117/940 (12.4)  193/940 (20.5)  60/940 (6.4)  46/940 (4.9)  15/940 (1.6)  36/940 (3.8)  74/940 (7.9)  43/940 (4.6)  54/940 (5.7)  25/940 (2.5)  38/940 (4.0) | Lacidipine  120/942 (12.7)  129/942 (13.7)  90/942 (9.6)  135/942 (14.3)  38/942 (4.0)  35/942 (3.7)  62/942 (6.6)  32/942 (3.4)  42/942 (4.5)  18/942 (1.9)  33/942 (3.5) | 0.98 (0.77-1.24)  1.50 (1.22-1.84)  0.67 (0.49-0.91)  0.34 (0.25-0.47)  0.40 (0.22-0.71)  1.03 (0.65-1.63)  1.20 (0.86-1.66)  1.35 (0.86-2.11)  1.29 (0.87-1.91)  1.39 (0.76-2.53)  1.15 (0.73-1.82) | NR  NR  NR  NR  NR  NR  NR  NR  NR  NR  NR |

.

AEs: adverse events; SAEs: serious adverse events; HCT: hydrochlorothiazide; MI: Myocardial Infarction; CVD: cardiovascular disease; CHD: coronary heart disease; HF: heart failure; CHF: congestive heart failure; TIA: transient ischaemic attack; T: Thiazide; C: Comparator; CI: confidence interval; HR: hazard ratio; OR: Odds ratio; RR: risk ratio; NR: Not Reported; *number of patients with the outcome/total patients unless stated otherwise, unreported counts/rates were derived from available data where possible; ^$^Calculated risk ratio unadjusted for covariates, zero cell adjustment applied where relevant; ^#^Reported comparison, adjusted for covariates if available
