# Supplementary material for: Thiazides in the management of hypertension in older adults – a systematic review
Source: BMC Geriatr. 2017 Oct 16;17(Suppl 1):228. doi: 10.1186/s12877-017-0576-3 (PMC5647553; doi:10.1186/s12877-017-0576-3)
Supplement: Supplementary file 5 — Recommendations developed for thiazide use in adults aged ≥65 years. (DOCX 15 kb) [file 12877_2017_576_MOESM5_ESM.docx]

**Additional file 5: Table S4: Recommendations developed for thiazide use in adults aged ≥65 years**

| **Recommendations** | **Strength of the recommendation** | **Quality of the evidence** | **Type of evidence** |
| --- | --- | --- | --- |
| It is suggested to reduce high dose of thiazides for the management of hypertension because high dose (50 to 90 mg/day hydrochlorothiazide or equivalent) may be less effective in reducing mortality and coronary artery disease and may be associated with higher risk of gout compared to other antihypertensive medication including low thiazide doses. *If the patient has also heart failure, please take the symptoms of heart failure additionally into account.* | Weak  Reason: Although the outcomes were considered to be critical for making a recommendation, the quality of the evidence was low which led to a weak strength of the recommendation | Low  It was considered to downgrade the quality of the evidence because there were study limitations (1 observational study, 1 secondary analysis of a RCT) and indirectness (SR reporting on younger populations, different types of thiazides) | Observational study (Gurwitz et al.1997), secondary analysis of a RCT (Fletcher et al.1991  Additional references of interest:  STOPP list (Gallagher 2008), 1 guideline (American College of Rheumatology Guidelines for Management of Gout)  1 SR (Wright et al. 1999) |
| It is suggested to discontinue both drugs belonging to the combination of hydrochlorothiazide and triamterene in older adults over the age of 80 because the benefits of this treatment may not be established in this age group when compared with placebo and because it may be associated with the development of gouty arthritis, especially in men with high serum uric acid and creatinine levels. | Weak  Reason: Although the outcomes were considered to be critical for decision making, the quality of the evidence was low which led to a weak strength of the recommendation | Low  It was considered to downgrade the quality of the evidence because there were study limitations (1 secondary analysis of a RCT) and imprecise results (few patients and outcome events) | 1 RCT (Staessen 1991, Amery 1986) |
| It is suggested to discontinue the combination of benazepril and hydrochlorothiazide and use the combination of benazepril and amlodipine instead. The combination of benazepril and hydrochlorothiazide for patients over the age of 65 with hypertension may be associated with an increase in deaths from cardiovascular causes combined with other cardiovascular events (specifically, nonfatal myocardial infarction, stroke, hospitalisation for unstable angina, coronary revascularisation and resuscitation after cardiac arrest) in this population compared with the treatment combination of benazepril and amlodipine. | Weak  Reason: Although the outcomes were considered to be critical for decision making, the quality of the evidence was low which led to a weak strength of the recommendation | Low  It was considered to downgrade the quality of the evidence because of risk of bias and lack of other RCTs supporting the evidence | 1 RCT (Jamerson et al.) |
